# Acute Leukaemia following Dengue Infection in Nepalese Patients: A Report of Two Cases

**DOI:** 10.1155/2024/8747138

**Published:** 2024-07-28

**Authors:** Anushka Agrawal, Pratik Lamichhane, Rituraj Baral, Sabin Thapaliya

**Affiliations:** ^1^ Maharajgunj Medical Campus Institute of Medicine, Kathmandu, Nepal; ^2^ Department of Pathology Tribhuvan University Teaching Hospital, Kathmandu, Nepal; ^3^ Department of Internal Medicine Tribhuvan University Teaching Hospital, Kathmandu, Nepal

## Abstract

Dengue is a mosquito-borne, acute febrile illness caused by dengue viruses. The association between hematological malignancies and dengue infection is obscure, and the literature on this occurrence is also limited. We report two cases of acute leukaemia following dengue infection in a recent outbreak in Nepal. Our case reports suggest a possible association of acute leukaemia with dengue infection. The relationship should be explored further with observational studies.

## 1. Introduction

Dengue is a mosquito-borne, acute febrile illness caused by an infection with one of the four serotypes of dengue viruses. It is transmitted by the bite of female *Aedes aegypti* or *Aedes albopictus* mosquitoes [1]. There is a wide spectrum of presentation following dengue infection, ranging from mild symptoms to severe, life-threatening symptoms. WHO published a revised classification of dengue in 2009 with the following categories: dengue without warning signs, dengue with warning signs, and severe dengue. A vast majority of dengue cases are asymptomatic and, hence, underreported. Dengue epidemics follow a seasonal pattern, with peaks during and after the rainy season. Dengue is endemic in many countries, with an estimated 100–400 million infections occurring worldwide each year. About 3.9 billion people are at risk of infection with dengue viruses [2, 3]. Dengue is found in tropical and subtropical climates worldwide, mostly in urban and semiurban areas. Nepal reported its first dengue case in 2004 from the Terai region, with massive outbreaks all over the country in 2010, 2013, and 2016. The rapid spread of dengue in Nepal is believed to be due to the spread of vectors, rapid urbanization, increased travel, and changing climatic conditions [4–6].

Acute myeloid leukaemia is a hematological malignancy which involves the proliferation, clonal expansion, and differentiation arrest of myeloid progenitor cells in the bone marrow. There is accumulation of leukaemia blasts and other immature forms in the bone marrow and peripheral blood. It is the most common acute leukaemia in adults, with about 80% of cases in this age group. AML has been associated with various aetiologies like environmental factors (radiation, tobacco, and chemotherapy), genetic factors, and other hematological disorders like myelodysplastic syndrome, paroxysmal nocturnal hemoglobinuria, and aplastic anemia [7, 8]. Acute lymphoblastic leukaemia and lymphoma are classified into B-cell ALL/LBL and T-cell ALL/LBL, depending on the cell lineage. The worldwide annual incidence of ALL is estimated to be around 1–5 cases per 100,000 people, and among these, about two-thirds are of the B-cell phenotype. It is more common in children, with a second peak occurring in adults older than 60 years. The aetiology of B-cell ALL is unknown but is mostly associated with ionising radiation, infection, single gene mutations, and trisomy 21 [9].

Certain viruses such as human T-cell lymphoma/leukaemia virus-1 (HTLV-1) and Epstein–Barr virus (EBV) have been identified as the risk factors of acute leukaemias. These viruses are known to infect the hematological precursor cells and deregulate their differentiation leading to malignant transformation of the affected cells [10]. Additionally, viral antigens have been implicated in upregulation of unchecked cellular proliferation owing to the gain-of-function mutations in various protooncogenes [11]. However, the association between hematological malignancies and dengue infection is not clear yet. Here, we present two uncommon cases of acute leukaemias following dengue infection in adult Nepalese women.

## 2. Case Presentation

### 2.1. Patient 1

A 64-year-old nonsmoker, nonalcohol consumer lady with a history of rheumatoid arthritis presented to TUTH emergency with intermittent fever (maximum temperature-102°*F*), chills and rigor, decreased appetite, and generalized weakness for 10 days. She also complained of appearance of multiple bluish patches over the body. She had no known sick contacts and denied of known comorbidities. On examination, she had a temperature of 100.5° *F*. She was tachycardiac with a pulse rate of 104 beats per minute. Her blood pressure was 90/60 mmHg on sitting position. Her general physical examination was within normal limits except for the presence of pallor. Her initial lab investigations showed leucocytosis (total leukocyte count-51,400 cells/mm^3^), anemia (hemoglobin-8.2 *g*/dl), and low platelet count (10,8000 cells/mm^3^). Additionally, the urine routine and microscopic examination showed plenty of pus cells. Additional tests to detect the infectious causative agents were performed. Out of few tests sent, only the dengue NS1 antigen test was positive, whereas dengue IgM and IgG tests were negative. Likewise, the IgM ELISA tests for leptospirosis and brucellosis were also negative. A peripheral blood smear was performed that showed anisopoikilocytosis with microcytic hypochromic RBCs, pencil cells, and polychromasia. Moreover, leucocytosis was observed with 70% blast cells that were 2-3 times the size of mature lymphocytes with scant to moderate amount of agranular cytoplasm, oval to cleaved nuclei, and fine chromatin. The platelets were large to giant in size with reduced number (6-7/OIF). The details of lab investigations for the patient are provided in Table 1.

**Figure 1:**
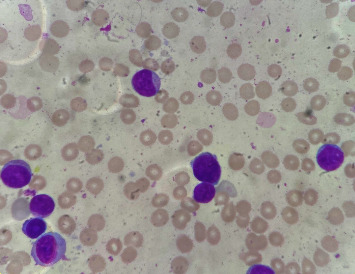
Cytological examination of bone marrow aspiration smear showing myeloblasts of intermediate size with scant to moderate cytoplasm (oil immersion field/1000x).

The positive dengue NS1 test led to the initial diagnosis of dengue fever, but the blood investigations were suggestive of acute leukaemia. Hence, a bone marrow aspiration and flow cytometry was done which revealed hypercellular marrow with erythropoiesis and leucopoiesis. The cytological examination of bone marrow aspirate showed blasts of intermediate size having scant to moderate cytoplasm, occasional granules, fine chromatin, and 1–3 prominent nucleoli (Figure 1). Flow cytometric analysis revealed a CD45 dim population (81% of the total) of atypical cells/blasts which were positive for CD34 (subset), CD117 (precursor markers), HLA-DR, CD13, CD33, CD38, MPO, CD11c, and CD36. These findings were consistent with acute myeloid leukaemia. During her stay at the hospital, the patient remained febrile and also developed cough. The daily blood investigations showed rising trend in WBC counts which peaked to about 112,000 cells/mm^3^ and then went on a decreasing trend during her course of stay. Hemoglobin levels ranged between 5.1 and 8.6 *g*/dl, and platelet counts ranged from 10,000 to 59,000 cells/mm^3^ during her stay. She received multiple blood transfusions with three pints of packed RBC and four pints of platelets in total. The patient was managed with intravenous paracetamol, antibiotics, and azacitidine and was being planned for venetoclax. Meanwhile, the patient developed respiratory distress, hypotension (76/39 mmHg), and fall in GCS for which she was shifted to ICU. The patient was intubated, started on IV fluids, and CPR was started when she developed an asystole. The CPR could not bring return of spontaneous circulation, and hence, the patient was declared dead.

### 2.2. Patient 2

A 57-year-old lady with a history of Type 2 diabetes mellitus, hypertension, and hypothyroidism presented to TUTH emergency with fever (maximum temperature:100° *F*), chills and rigor, generalized weakness, and headache for last three days. She also gave a history of similar illness in her family members. She was a nonsmoker and did not consume alcohol. On examination, all her vitals were stable except for a high temperature of 100°F. Other clinical examinations were within normal limits. The laboratory investigations showed leucopenia, thrombocytopenia, deranged liver function tests, and hyponatremia. The dengue NS1 test was positive, whereas the dengue IgM and dengue IgG tests were negative. The details of lab investigations for the patient are provided in Table 1. She was then admitted in our center with a diagnosis of dengue fever with warning signs. She was managed conservatively with intravenous fluids and antipyretics and discharged after a week following resolution of her condition.

**Figure 2:**
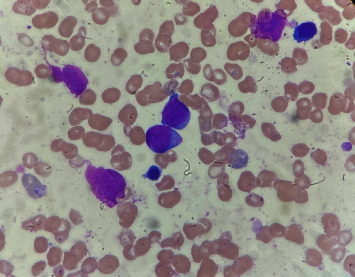
Cytological examination of bone marrow aspiration smear showing large sized lymphoblasts of acute lymphoblastic leukemia (oil immersion field/1000x).

She was readmitted to our center a month later due to anemia (hemoglobin-7.9 *g*/dl), persistent thrombocytopenia, and leukocytosis (TLC- 25,100 cells/mm^3^). The repeat dengue IgM test was weak positive, and dengue IgG test and NS1 tests were also positive. During her second admission at the hospital, she also developed a neck swelling with multiple enlarged lymph nodes in bilateral submandibular region and level 5 region as per ultrasound of the neck. These findings were suggestive of tubercular lymphadenitis and a differential of lymphoma was kept. Hence, a fine needle aspiration cytology of cervical lymph node was done which showed mixed population of lymphoid cells at various stages of maturation and a few histiocytes and mast cells likely to be a reactive lymph node. Additionally, sputum analysis for *acid fast bacilli of tuberculosis* was found to be negative. A peripheral blood smear was done which showed 60% blast cells suggestive of acute leukaemia. Additionally, bone marrow aspiration showed blasts cells (in the center of the field) having moderate amount of basophilic cytoplasm, round nuclei with fine cheomatin, and 2-3 prominent nucleoli (Figure 2). The flow cytometric analysis was consistent with precursor B acute lymphoblastic leukaemia/lymphoma (CD10 positive, CD34 positive, TdT positive). A BCR-ABL1 translocation assay quantitative real-time RT-PCR was done where the minor BCR-ABL1/ABL1 transcript was detected to be 18.42%. She also developed pleural effusion, and the fluid analysis showed lymphocyte-rich effusion suggestive of lymphoproliferative disorder. The patient is still admitted. The treatment is ongoing at the time of completion of writing of the report.

## 3. Discussion

In this case report, we described two cases of hematological malignancy who presented to us following an initial diagnosis of severe dengue infection. Both cases were apparently well before and were admitted with complaints of fever with chills and rigor and dengue NS1 antigen test positive. However, after the primary diagnosis of dengue, one of the patients developed leucocytosis, anemia, and thrombocytopenia, whereas the other developed leucopenia, anemia, and thrombocytopenia. The peripheral blood smears and bone marrow aspiration then revealed blast cells of myeloid lineage and lymphoblastic lineage in patient 1 and patient 2, respectively.

There have been a few reports of leukaemias mimicking dengue infection, but a clear association is yet to be explored [12, 13]. The dengue nonstructural protein 1 (NS1) is an essential component of viral replication which can be detected in serum samples of infected patient since day 1 of symptom onset and is used as an early diagnostic tool [14]. Nonstructural proteins are dimer proteins with the molecular weight ranging from 46 to 55 kDa depending on the extent of glycosylation. This dimer protein has two parts: an intracellular part which is central to viral replication and the membrane-bound part that is secreted and elicits immune response [15].

Patient 1 who presented with fever and headache had a dengue NS1 antigen positive, but IgM and IgG were negative. However, the prolonged fever for a month was an unusual presentation of dengue, and further investigations led to a final diagnosis of acute myeloid leukaemia. A few cases of acute leukaemias have been diagnosed in patients who presented with fever and dengue NS1 positive as per the reports from other parts of the world [12, 16]. Supat et al. [12] suggested a possibility of false-positive NS1 antigen tests in patients with leukaemia. There are no reports yet mentioning any definite cause for false positive NS1 in hematological malignancies. However, a probable cause that has been postulated is that the malignant cells undergo rapid apoptosis which releases intracellular proteins that may bear homology to the dengue NS1 antigen. As in this case, since RT-PCR was not performed for dengue virus, dengue infection could not be ruled out completely. Hence, the possibility of the false-positive NS1 dengue test still remains in regards to both of our cases. We consider this as one of the limitations in case management in resource-constrained setting like ours where RT-PCR facility for dengue infection is not available widely.

Patient 2 also presented with a history of fever with pancytopenia and a positive dengue NS1 antigen test which was followed by a positive IgM and IgG test after few weeks. A dengue RT-PCR was not done in this case either due to unavailability of testing services. Hence, a definite evidence of dengue infection could not be proved. Detailed blood and marrow investigations led to the diagnosis of B-cell ALL. The association between dengue infection and hematological malignancy is still unknown. Many viruses with well-known association as a causative agent of hematological malignancies such as Epstein–Barr virus [17] and HIV [18], but evidence of similar causative association between dengue and leukemia has not been proved. However, reports of dengue causing haemophagocytic syndrome leading to blood cytopenia are widely available in the literature [19]. This condition is called virus-associated haemophagocytic syndrome (VAHS) and is characterised by a systemic proliferation of non-neoplastic histiocytes and haemophagocytosis [20]. It is hypothesized that viral infection causes an aberrant immune response in those who are susceptible, resulting in hyperactivation of Th1 helper cells, macrophage growth, and large-scale cytokine release. The clinical and biochemical symptoms of VAHS may be brought on by the ensuing hypercytokinaemia [19, 21]. Therefore, pancytopenia observed in patient 2 could be explained by the possibility of development of VAHS following dengue infection.

## 4. Conclusion

Our case reports suggest a possible coincidental association of acute leukemia with dengue infection. The association between dengue infection and leukaemia can be demonstrated after concrete evidence of infection has been established with RT-PCR. It is equally important for physicians to remember that acute leukaemias can give rise to false positive NS1 antigen test. The relationship beyond coincidental association can be verified with the availability of further reports on similar occurrences.

## Figures and Tables

**Table 1:** Details on lab investigation of both patients.

Investigations	Patient 1	Patient 2
*Complete blood count*		
Total leukocyte count (cells/mm^3^)	51,400	1,300
Hemoglobin (g/dl)	8.2	11.2
Platelets (cells/mm^3^)	108,000	32,000
INR	1.3	1.08

*Renal function tests*		
Urea (mmol/L)	2.7	4.4
Creatinine (mmol/L)	50	69
Uric acid (mmol/L)	150	437

LDH (U/L)	893	N/A

*Liver function tests*		
AST (U/L)	35	68
ALT (U/L)	15	45

Dengue IgM test	Negative	Negative

Dengue IgG test	Negative	Negative

Dengue NS1 antigen test	Positive	Positive

Brucella ab test	Negative	N/A

Leptospira IgM test	Negative	N/A

Leptospira IgG test	Negative	N/A

Sputum AFB test	N/A	Not detected

N/A: not available.

## Data Availability

The data described in the article can be obtained upon reasonable request to the authors.
